# Population genetics and external proficiency testing for HLA disease associations

**DOI:** 10.3389/fgene.2023.1268705

**Published:** 2023-10-23

**Authors:** Frantisek Mrazek

**Affiliations:** HLA laboratory, Department of Immunology, University Hospital, Olomouc, Czechia

**Keywords:** external proficiency testing, HLA, disease association, polymorphism, genetic susceptibility

## Abstract

Numerous associations of HLA variants with susceptibility to diseases, namely, those with an immunopathological component, have been described to date. The strongest HLA associations were incorporated into the standard algorithms for the diagnostics. Disease-associated HLA variants are routinely detected by various techniques including DNA-based assays. For the identification of HLA markers or their combinations with the highest diagnostic value and those with frequent clinical indications (e.g., HLA-B*27, -B*57:01, -DQ2/-DQ8, -DQB1*06:02), diagnostic tests that focus on a single or limited number of specific HLA antigens/alleles, have already been developed; the use of complete typing for particular HLA loci is a relevant alternative. Importantly, external proficiency testing (EPT) became an integral part of good laboratory practice for HLA disease associations in accredited laboratories and not only supports correct “technical” identification of the associated HLA variants, but also adequate interpretation of the results to the clinicians. In the present article selected aspects of EPT for HLA disease associations related to population genetics are reviewed and discussed with the emphasis on the optimal level of HLA typing resolution, population-based differences in disease associated HLA alleles within the allelic group, distribution and linkage disequilibrium of HLA alleles in particular populations and interpretation of the presence of less common HLA variants/haplotypes. In conclusion, the laboratories that perform and interpret the tests to the clinicians, producers of the certified diagnostics and EPT providers should consider, among others, the genetic characteristics of the populations in order to optimise the diagnostic value of the tests for disease-associated HLA variants.

## 1 Introduction

Early after the discovery of the polymorphic human major histocompatibility complex (MHC) encoding for the human leukocyte antigens (HLA), the first reports on the association of particular HLA variants with diseases were published (Brewerton et al., 1973; Woodrow, 1973; Thorsby, 2009). These studies were based on comparisons of the presence of HLA variants between the patients and the age/sex/ethnically matched control subjects. Such pioneer empirical observations were not usually accompanied by any data explaining the causal mechanism of the associations. Nevertheless, it is important to remember, that exact mechanisms of many associations between the HLA system and diseases are not currently clear, despite several plausible hypotheses being available. An enormous number of HLA associations have thus far been described based on both a candidate gene and genome-wide approach. Particularly in autoimmune diseases, the variability of the MHC genes at the short arm of chromosome 6 substantially contributes to the genetic susceptibility of these conditions, e.g., in type 1 diabetes or rheumatoid arthritis (Wellcome Trust Case Control Consortium, 2007). Of the confirmed HLA disease associations, those with the strongest relationship with the diseases were integrated as part of the complex diagnostics of the associated diseases, namely, due to their high negative predictive value (Altman and Bland, 1994; Tye-Din et al., 2015).

Great effort has been dedicated to development of rapid and reliable laboratory procedures to identify variants with the highest value for the assessment of disease susceptibility under reasonable costs (Rouvroye et al., 2019). HLA variants with the most frequent clinical indications are represented, e.g., by HLA-B27 in rheumatology (Dequeker et al., 1978), HLA-DQ2/-DQ8 for celiac disease (Tye-Din et al., 2015) or HLA-DQB1*06:02 in narcolepsy (Tafti et al., 2014). HLA tests that support (contra) indications of the specific pharmacotherapy, such as HLA-B*57:01 for abacavir application in HIV treatment (Mallal et al., 2002) or HLA-A*02:01 for indication of targeted therapy of metastatic uveal melanoma by tebentafusp (Nathan et al., 2021) have recently been established. It is worth remembering, that there are further enormously important applications of the HLA disease association studies beyond direct detection of HLA variants for diagnostics reasons, namely, for understanding the molecular pathogenesis of complex diseases with immunopathological component (e.g., Ciacchi et al., 2022), but this area is out of scope of this rather practically oriented minireview.

Clinical application of the HLA tests for the diagnosis of associated diseases must always be based on the strong scientific evidence of a relationship between the HLA variant (or combination of HLA variants) and the disease. Detailed and independently confirmed data of the associated HLA variant should optimally be available for particular ethnics/populations, including the frequency of the HLA variant in the general population, relative risk of the condition conferred by the variant, the specificity, sensitivity, positive/negative predictive values, population attributable risk and further epidemiological parameters of the association. Because of the great complexity of the polymorphic HLA system, in the present article we focus on the selected aspects of population genetics in the HLA disease associations that may be relevant for design, organisation, interpretation and outcome of the EPT schemes in order to not only support correct “technical” identification of the associated HLA variants, but also adequate interpretation of the results by laboratories and their application in the diagnostic process.

## 2 HLA polymorphism, population genetics and disease associations

Currently known global HLA polymorphism is enormous (Barker et al., 2023) and may be demonstrated, e.g., by the fact that for the substantial percentage of patients seeking a donor for haematopoietic stem cell transplantation, no fully HLA matched unrelated donor can actually be found among the more than 41 million currently available donors in World Marrow Donor Association database (WMDA, 2023). The vast amount of data shows that global distribution of HLA alleles strongly differs in various ethics and populations (Gonzalez-Galarza et al., 2020; Arrieta-Bolaños et al., 2023). This fact has important consequences for identification, evaluation, clinical application and interpretation of associations of HLA variants with diseases. There is clear evidence that the distribution of HLA alleles in the concrete population may directly affect the prevalence and clinical manifestation of HLA associated diseases (Yazici et al., 2018). In cases of tight disease association with the common HLA variant in the population, the frequency of the variant correlates with the occurrence of the disease and confers high values of population attributable risk for the condition.

The impact of population genetics to the HLA disease association studies is, however, complex and may not be reduced to the relationship between the prevalence of the disease and frequency of the predisposing HLA variant in the population. It has been shown that different alleles within the same HLA allelic groups or even those from different allelic groups and HLA loci, may confer susceptibility to the same disease, e.g., to Vogt-Koyanagi-Harada disease, in various populations (Huang and Brown, 2022). Furthermore, linkage disequilibrium (LD, see Section 3) observed in the MHC region is reflected in different haplotypes characteristic for the particular populations, including those possessing predisposing HLA or other linked causal variants. Importantly, the relationship between the HLA risk variants and diseases may be modified by the polymorphism outside the HLA genes, and even outside the MHC genetic region. The role of such gene-gene interactions between the variants from different genes (epistatic effect) depends, among others, on the overall occurrence of these variants in particular populations. There is, for example, a well-known epistatic effect of the *ERAP1* (endoplasmic reticulum aminopeptidase 1) gene to the HLA-B51 association with Behçet’s disease (Kirino et al., 2013). In this case the specific *ERAP1* alleles strengthen the effect of HLA-B51 on disease susceptibility. This fact supports the implication of the HLA-B51 associated peptidome in the pathogenesis of Behçet’s disease. Finally, both qualitative and quantitative patterns of the associations between HLA variants and diseases or conditions are modified by, often unknown, environmental factors and disease triggers that may substantially vary between the regions and populations (Sparks and Costenbader, 2014).

## 3 Linkage disequilibrium in MHC region

There is no doubt that linkage disequilibrium (LD), defined as nonrandom association between particular alleles at linked loci (Slatkin, 2008), is very common within the MHC genetic region at chromosome 6p and is responsible for the occurrence of numerous HLA haplotypes often characteristic for particular populations (Creary et al., 2021). From the viewpoint of the search for HLA disease associations, it was the strong LD in the HLA region that enabled substantial proportion of initial observations of the relationship between the HLA polymorphism and diseases, despite these studies only identifying the HLA marker in LD and not the causal HLA variant. On the other hand, tight LD in the HLA region complicates identification of the HLA or non-HLA variants really causal for the disease (Moutsianas and Gutierrez-Achury, 2018). Together with the enormous development of methods for HLA typing, the conduction of large candidate gene and genome-wide association studies from various populations followed by fine mapping of local polymorphisms within the HLA and nearby loci, supported by accompanying functional data, enabled the specification of causal HLA variants and haplotypes for numerous diseases (Gutierrez-Achury et al., 2015; Sciurti et al., 2018). Due to the LD in the HLA system, one can observe a long-term history of the evolving knowledge on many HLA associations with particular diseases. For example, currently well-documented association of celiac disease with the presence of HLA-DQ2.5 heterodimer (encoded in “cis” configuration by DQB1*02:01 and DQA1*05:01 alleles at the same HLA haplotype) (Tye-Din et al., 2015) was originally identified as an association with HLA-B8 (Evans, 1973), followed by HLA-DR3 (Ek et al., 1978); both these HLA antigens are encoded on the same and very common HLA haplotype together with DQB1*02:01 and DQA1*05:01 alleles. Further historical examples of HLA associations originally identified based on LD in the MHC region are listed in Table 1. From the practical point of view, HLA laboratories are sometimes faced with requirements to conduct typing of the HLA variants already obsolete for particular disease associations based on older literary reports. The example is provided in the guidance to the current Standards for Accredited Laboratories of the American Society for Histocompatibility and Immunogenetics (ASHI, 2023) where laboratories testing for narcolepsy risk would be expected to type for DQB1*06:02 and not for DRB1*15. In such cases, and if the laboratory registers a requirement for an unusual HLA test for particular disease, it is recommended to individually consult those indications with the clinicians in order to ensure the optimal diagnostic value of the test, and its proper interpretation, and use that as an opportunity for physician education.

**TABLE 1:** Linkage disequilibrium (LD) in HLA disease association studies: historical examples of evolving knowledge on HLA variants associated with particular diseases based on LD.

HLA associated disease	Associated HLA variants	References
Celiac disease	HLA-B8	Evans (1973)
	HLA-DR3	Ek et al. (1978)
	HLA-DQ2	Corazza et al. (1985)
	HLA-DQ2.5 heterodimer	Vartdal et al. (1996)
Type 1 diabetes	HLA-B8	Cudworth and Woodrow (1975)
	HLA-DR4	Farid et al. (1979)
	HLA-DQ8	Owerbach et al. (1989), Baisch et al. (1990)
Psoriasis (psoriatic arthritis)	HLA-B13	Karvonen et al. (1976)
	HLA-B17	Gunn et al. (1979)
	HLA-Cw6	Tsuji et al. (1979)
Narcolepsy (cataplexy)	HLA-B7	Seignalet and Billiard (1984)
	HLA-DR2	Juji et al. (1984)
	HLA-DQB1*06:02	Mignot et al. (1994)

## 4 Methodical aspects of HLA disease association studies

Historically, numerous tests based on various methodical principles were developed to identify HLA variants associated with diseases. Among the techniques that use antibodies specifically targeting the HLA molecules expressed on the cell surface, the lymphocytotoxicity test (complement-dependent cytotoxicity, CDC) or flow cytometry (Albrecht and Müller, 1987) are widely used to date. Nevertheless, these approaches are limited by the level of resolution of the HLA variants, because they are usually able to provide information at “low resolution” level (e.g., the presence of HLA-B27). Such disadvantage is eliminated by DNA-based assays that recognise HLA polymorphism directly on the HLA genes and, therefore, any level of HLA resolution reliable for particular disease association may be obtained. In order to detect HLA variants associated with disease susceptibility, one approach aims at identification of the single associated HLA variant in the subject; such tests are particularly used for their lower costs. The second approach is based on the evaluation of the disease-associated HLA variants from the complete HLA type of the individual at particular HLA locus (loci). The knowledge of the complete HLA type at relevant locus may be preferred in situations when the information on the heterozygous/homozygous status of the HLA allele provides important additional information for the disease susceptibility (i.e., risk stratification of the subjects based on the gene-dose effect) (Megiorni and Pizzutti, 2012). In general, any laboratory technique intended for identification/typing of disease-associated HLA variants, both commercially available or “in house” (when allowed for particular purpose) should undergo a validation/verification process in order to demonstrate that it is able to provide reliable and reproducible results with adequate diagnostic value.

## 5 HLA typing resolution

In order to characterise HLA type of the individual for different clinical/research purposes, several levels of HLA typing resolution by DNA techniques have been established (Nunes et al., 2011). “Low resolution” typing identifies HLA allelic groups (e.g., HLA-B*27) and, in the majority of cases, reflects the level of typing obtained by antibody-based techniques such as CDC (e.g., HLA-B27). By contrast, “high resolution” (e.g., HLA-B*27:05P) and “allelic resolution” (e.g., HLA-B*27:05:02:01) provide more detailed information on HLA variants within allelic groups. Before the arrival of modern DNA-based assays, a limited number of elemental HLA “specificities” could be recognised by serological or antibody/cell–based techniques such as CDC. Nevertheless, these techniques enabled discovery of numerous currently known HLA disease associations.

Based on the results from genetic-association studies, the required level of HLA typing resolution was defined for appropriate assessment of the genetic susceptibility conferred by particular HLA variants. For example, information about the presence of HLA-DQB1*02/-DQA1*05 at low resolution level is usually accepted for the interpretation of the test for predisposition to celiac disease (heterodimer DQ2.5). In contrast, a low resolution level (HLA-DQB1*06) is not informative for the evaluation of the genetic risk to narcolepsy, where the HLA-DQB1*06:02 allele is strongly susceptible but another common DQB1*06:03 variant confers protection from the disease (Tafti et al., 2014). Similarly, only patients carrying HLA-A*02:01 and not those with other HLA-A*02 variants are eligible, from the genetic point of view, for the therapy of metastatic uveal melanoma by tebentafusp (Table 2; Nathan et al., 2021).

**TABLE 2:** Distribution of HLA variants associated with diseases/conditions in various populations.

HLA association	HLA allelic group	Associated HLA allele(s)	Population*	Sample size	HLA allelic group (%)	Associated HLA alleles in the allelic group (%)	Associated HLA alleles carriers (%)
Ankylosing spondylitis	HLA-B*27	B*27:02 B*27:04 B*27:05 B*27:07	Germany DKMS - German donors	3,456,066	4.5	98.2	8.6
			USA NMDP Vietnamese	43,540	2.5	55.6	2.8
			USA NMDP Filipino	50,614	3.4	29.4	2.0
Tebentafusp indication	HLA-A*02	A*02:01	Germany DKMS - German donors	3,456,066	29.6	95.9	48.7
			USA NMDP African American pop2	416,581	18.1	68.2	23.2
			Israel Morocco Jews	36,718	14.5	17.1	4.9
Narcolepsy (cataplexy)	HLA-DQB1*06	DQB1*06:02	Germany DKMS - German donors	3,456,066	25.7	50.4	24.2
			USA NMDP Hispanic South or Central American	5,764	17.5	42.7	14.4
			USA NMDP Vietnamese	1,032	10.8	10.8	2.3

The data (including designation of example populations*) was adopted from the publicly available Allele frequency net database (http://www.allelefrequencies.net/, Gonzalez-Galarza et al., 2020). For each population, frequency of the allelic group (e.g., HLA-A*02), percentage of susceptible allele(s) (e.g., HLA-A*02:01) out of all alleles in the allelic group, and calculated estimation of the carriers of the allele(s) associated with the condition are given.

For a very long time there was a consensus that the presence of HLA-B27 is strongly associated with the rheumatic diseases, such as ankylosing spondylitis (Brewerton et al., 1973) or Reiter’s syndrome (Woodrow, 1973). Antibody-based HLA typing techniques, such as CDC or flow cytometry, are therefore still in use for selected HLA disease associations. Accordingly, several EPT programs offer identification, e.g., of HLA-B27, by serological or antibody-based techniques (INSTAND, 2023). However, there is evidence that in addition to the HLA-B*27 alleles conferring the risk of the diseases (e.g., HLA-B*27:05, -B*27:02), other HLA-B*27 variants are protective (HLA-B*27:06, B*27:09) (Costantino et al., 2018). In recent years substantial differences in the distribution of susceptible/neutral/protective HLA-B*27 variants in various populations have been debated, including the fact that such variability affects the diagnostic value of the HLA-B27 test. Based on the publicly available population data on HLA polymorphism, the occurrence of HLA-B*27 protective alleles in European Caucasoid populations is rare (Table 2; Gonzalez-Galarza et al., 2020). In other words, almost each Caucasoid patient with the presence of HLA-B27 is a carrier of the HLA-B*27 risk allele. In contrast, in some Asian populations (e.g., Filipino, Vietnamese), susceptible HLA-B*27 alleles are much less frequent among all HLA-B*27 alleles (only approx. 30% in the Filipino population, Table 2). Therefore, the majority of patients of Filipino ancestry possessing the HLA-B27 antigen are not at increased genetic risk of the rheumatologic diseases mentioned above. From this point of view diagnostic value of the HLA-B27 defined at low resolution level may significantly differ in various populations. Accordingly, there is a possibility to consider the distribution of HLA-B*27 alleles in the population for which the laboratory provides the tests and, if appropriate, to apply identification of HLA-B*27 alleles at a high resolution level for all samples or those from subpopulations with a lower proportion of susceptible HLA-B*27 variants. From the same reason, EPT programs that will provide both levels of resolution in EPT for single HLA-B27 testing, i.e., for the presence of HLA-B27 and for the presence of particular susceptible HLA-B*27 alleles, may improve the overall value of HLA-B27 testing in clinical applications.

## 6 EPT in HLA disease association studies

EPT is considered to be an integral part of the HLA testing for disease associations. For example, according to the current standards for accreditation of laboratories by the European Federation for Immunogenetics (European Federation for Immunogenetics, 2020), the laboratory must document all tests performed for HLA disease association (diseases, spectrum of tested HLA variants) and participate in EPT for each performed investigation including the detection of single HLA variants for disease associations. The minimum of 10 samples per year should be tested and reported by the laboratory to the organiser of the EPT. If an EPT scheme or EPT workshop/trial for the identification of a specific HLA variant is not available, the laboratory must at least participate in an inter-laboratory exchange of samples. Very similar rules for EPT and testing for HLA disease associations are also valid for laboratories accredited by the American Society for Histocompatibility and Immunogenetics (ASHI, 2023). Furthermore, both the ASHI and Asia-Pacific Histocompatibility and Immunogenetics Association (APHIA) conduct their own proficiency testing programs in order to support improvements of overall quality of HLA testing (APHIA, 2023).

Complete typing for particular HLA loci, which is a relevant possibility for HLA disease association diagnostics, is covered by numerous EPT providers (see, e.g., the list of EPT Providers registered at EFI at https://efi-web.org/committees/ept-committee). On the other hand, the spectrum of commercially available certified tests that were designed for the detection of a single or limited number of associated HLA antigens or alleles continually grows. Unfortunately, relatively few EPT providers offer proficiency testing for the tests that target particular HLA variants and do not provide complete HLA typing. This problem could be partially resolved by the providers of EPT for standard (complete) HLA typing who will allow participants to only report the presence/absence of the HLA variant of interest, and such reports being analysed in a specific manner. This approach has already been established, e.g., by the ASHI proficiency testing program. This EPT scheme distributes the same EPT samples for both complete HLA typing and/or HLA-B27 detection based on the requirements of participating laboratories. In a similar model situation, EPT for any HLA-A*02:01 single allele test used for indication of the therapy by tebentafusp could be involved in the analysis of standard EPT for HLA typing, because the carriers of this variant represent up to 50% of the Caucasoid population. However, this approach will have a limitation for the less common HLA variants that may be absent in the EPT samples, except in case that the samples possessing those HLA variants will be specifically selected for the EPT.

To our knowledge established providers of EPT for HLA disease associations offer the schemes that follow the current knowledge and recommendations in HLA disease association studies and undergo regular evaluation by the EPT program steering committees. In order to properly reflect the developments in the field, EPT providers (e.g., the Institute of Hematology and Blood Transfusion in Prague) organise regular workshops with attendance of the experts in immunogenetics, opinion leading clinicians representing medical societies in particular fields of HLA disease associations (rheumatology, gastroenterology, neurology, etc.), manufacturers of the reagents and EPT participants. Such workshops also serve as a forum for interlaboratory harmonisation and updating the recommendations for the interpretation of the tests for HLA disease associations.

## 7 Conclusion

Identification of selected HLA variants or their combinations that predispose to the diseases or conditions were involved in the standard algorithms for the diagnostics of associated diseases, namely, for their negative predictive value. External proficiency testing (EPT) is considered to be an integral part of HLA testing for disease associations and not only supports the technical point of the tests, but also interlaboratory harmonisation in terms of their reporting, interpretation and outcome for the clinical application. In this article we focused on selected aspects of HLA disease association studies that are related to population genetics and may be relevant for the providers and participants of EPT for HLA disease associations. In general, all parties involved in the application of HLA tests for diagnostic purposes (clinicians indicating the tests, HLA laboratories, producers of the specific reagents and providers of EPT schemes) should be familiar with the current knowledge in the field including the standards and guidelines of the relevant medical societies. Regarding population genetics, both EPT providers and participants should consider HLA disease association at the level of resolution that reflects distribution of risk HLA variants in the relevant populations and ethnics, select HLA markers with the strongest informative value for the diagnostics (avoid obsolete HLA markers in LD), apply adequate methods and reagents for their identification, and, if beneficial for patients, interpret the results taking specific characteristics of the particular populations and ethnics (e.g., LD, allele frequency, distribution of genotypes, gene-gene and gene-environment interaction, epidemiological parameters) into account. In addition, the laboratories and EPT providers should have the mechanisms for how to interpret the presence of less common HLA variants/haplotypes without sufficient supportive data on their implication in disease susceptibility.
